# Double the Diagnosis: A Case Report of Dizygotic Twins With Meningomyelocele

**DOI:** 10.7759/cureus.70284

**Published:** 2024-09-26

**Authors:** Emily A Ina, Christopher C Roberts

**Affiliations:** 1 Medicine, Osteopathic Medical School, Nova Southeastern University Dr. Kiran C. Patel College of Osteopathic Medicine, Fort Lauderdale, USA; 2 Neurosurgery, Broward Health, Fort Lauderdale, USA

**Keywords:** dichorionic diamniotic twins, meningomyelocele, neural tube defects (ntds), neuro-surgery, pediatric hydrocephalus

## Abstract

The formation of neural tube defects, such as a meningomyelocele, is often sporadic and more common in monozygotic twin gestations but can be associated with folic acid deficiency and rarely occurs in dizygotic twins. In this case report, we present dizygotic twins born in the United States with meningomyeloceles. In addition, their neural tube defects were complicated by hydrocephalus and decreased muscle tone and movement in the lower extremities. Repair of the meningomyeloceles occurred 48 hours after birth with neurosurgical intervention, and placement of ventriculoperitoneal shunts was performed two weeks later once the neonates were of appropriate weight. This case report presents a rare occurrence of dizygotic twins with concurrent meningomyeloceles requiring post-natal surgical intervention to improve quality of life and decrease fatal complications of neural tube defects.

## Introduction

One of the most common congenital anomalies of the central nervous system is a meningomyelocele, with an incidence of approximately 0.2 to 0.4 per 1,000 live births in the United States [1]. The primary pathogenesis of meningomyelocele is the failed closure of the neural tube during embryonic development, resulting in a spinal cord that is exposed dorsally with the formation of a placode, a thickening of the embryonic ectoderm.

A strong genetic correlation is found in an estimated 60%-70% of patients with meningomyeloceles [2,3]. Additionally, there has been a higher concordance rate of neural tube defects in same-sex twins compared to opposite-sex twins with rates of 7.7% and 4.0% for monozygotic and dizygotic twins [2,3].

Symptoms of meningomyelocele frequently include physical disabilities such as paralysis and mobility difficulties, bladder and bowel dysfunction, hydrocephalus, and abnormal bone development including scoliosis or hip dislocation. The diagnosis of neural tube defects is typically detected through prenatal ultrasounds and maternal blood tests illustrating elevated alpha-fetoprotein levels. Diagnosis after birth can occur based on physical examination findings and confirmed with imaging studies, including magnetic resolution imaging (MRI) or computed tomography (CT) scans.

Treatment options for meningomyeloceles begin with neurosurgical intervention shortly after birth to repair the opening and protect the spinal cord. Once the spinal cord has been protected, ongoing care with a multidisciplinary approach involving neurology, orthopedic surgeons, urology, and physical therapy is vital. Further management and mitigation of complications such as hydrocephalus is crucial as the child continues to develop. While the prognosis varies, it is highly dependent on the extent of nerve damage and the presence of complications which ultimately impact one’s quality of life.

Meningomyelocele is a complex condition requiring comprehensive and multidisciplinary medical management, especially with neurological surgery and support. Given the rarity of occurrence, this case report aims to present dizygotic twins born with meningomyelocele defects and the management and outcome after neurosurgical intervention.

## Case presentation

In a dichorionic diamniotic twin pregnancy, each fetus, male and female, had their own placenta and amniotic sac and was conceived naturally in a 43-year-old African American woman (gravida 4, para 2, abortion 1). The twin pregnancy was complicated by breech presentation and prenatal diagnosis of neural tube defects in both twins with associated hydrocephalus.

The twins were born at 33 weeks of gestation by cesarean section after spontaneous rupture of membranes. The male (twin 1) weighed 2,155 g and the female (twin 2) weighed 1,555 g with stable vital signs but were later placed on high flow nasal cannula (2L) due to respiratory distress. On physical examination, both twins had increased head circumference with open anterior and posterior fontanelles with split sutures and notable frontal bossing. On neurological exam, the infants responded appropriately with normal and symmetric primitive reflexes including grasp and moro reflexes bilaterally. Additionally, decreased tone in lower extremities and unresponsive to stimuli with neural tube defects at the lumbar spine without active leakage of cerebrospinal fluid (CSF) were noted in both male and female neonates. No deformities were found in the upper extremities, abdomen, or genitalia.

**Figure 1:**
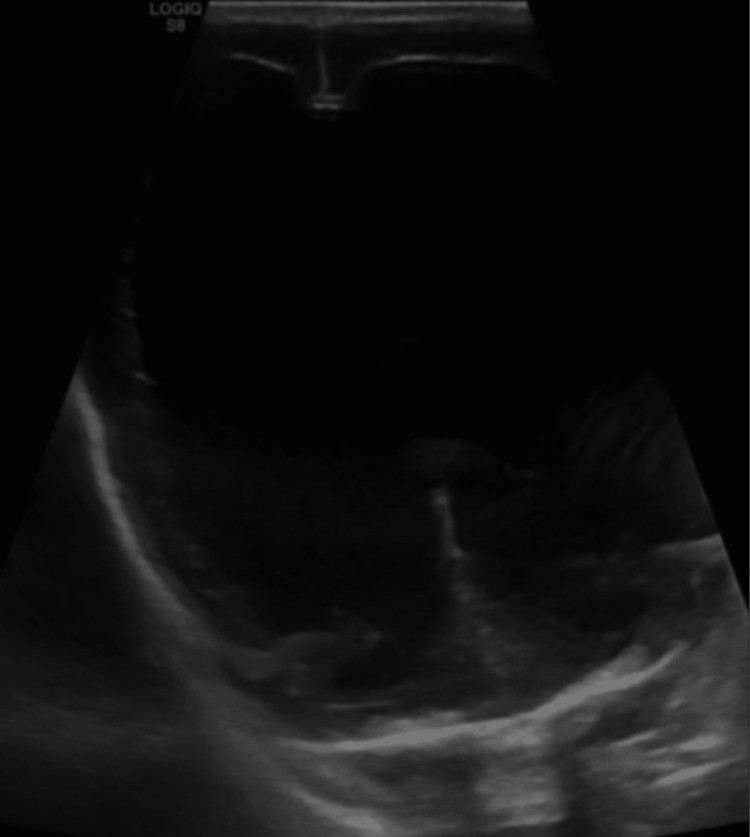
Ultrasonography of male neonatal head representing severe hydrocephalus with evidence of some thalamic tissue, but limited evaluation of brain structures.

**Figure 2:**
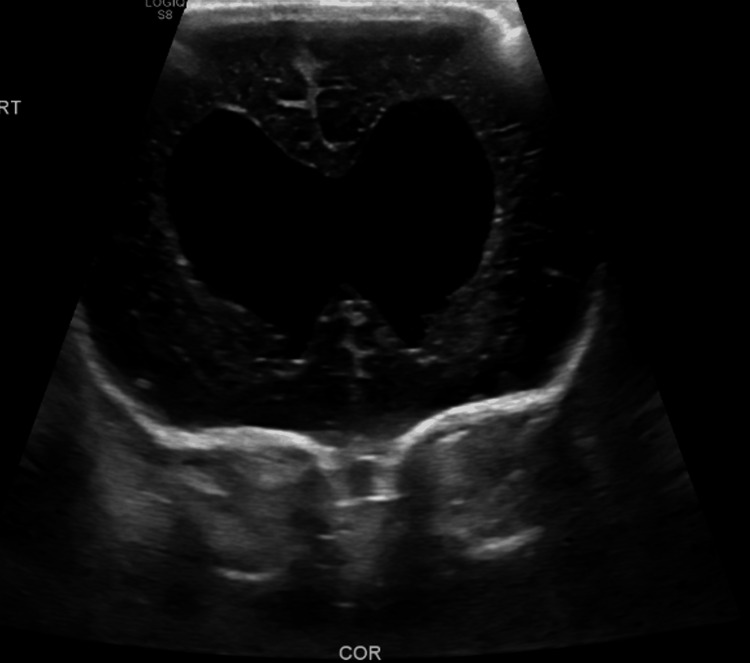
Head ultrasonography of twin female’s lateral ventricles illustrating batwing confirmation and ventriculomegaly.

**Figure 3:**
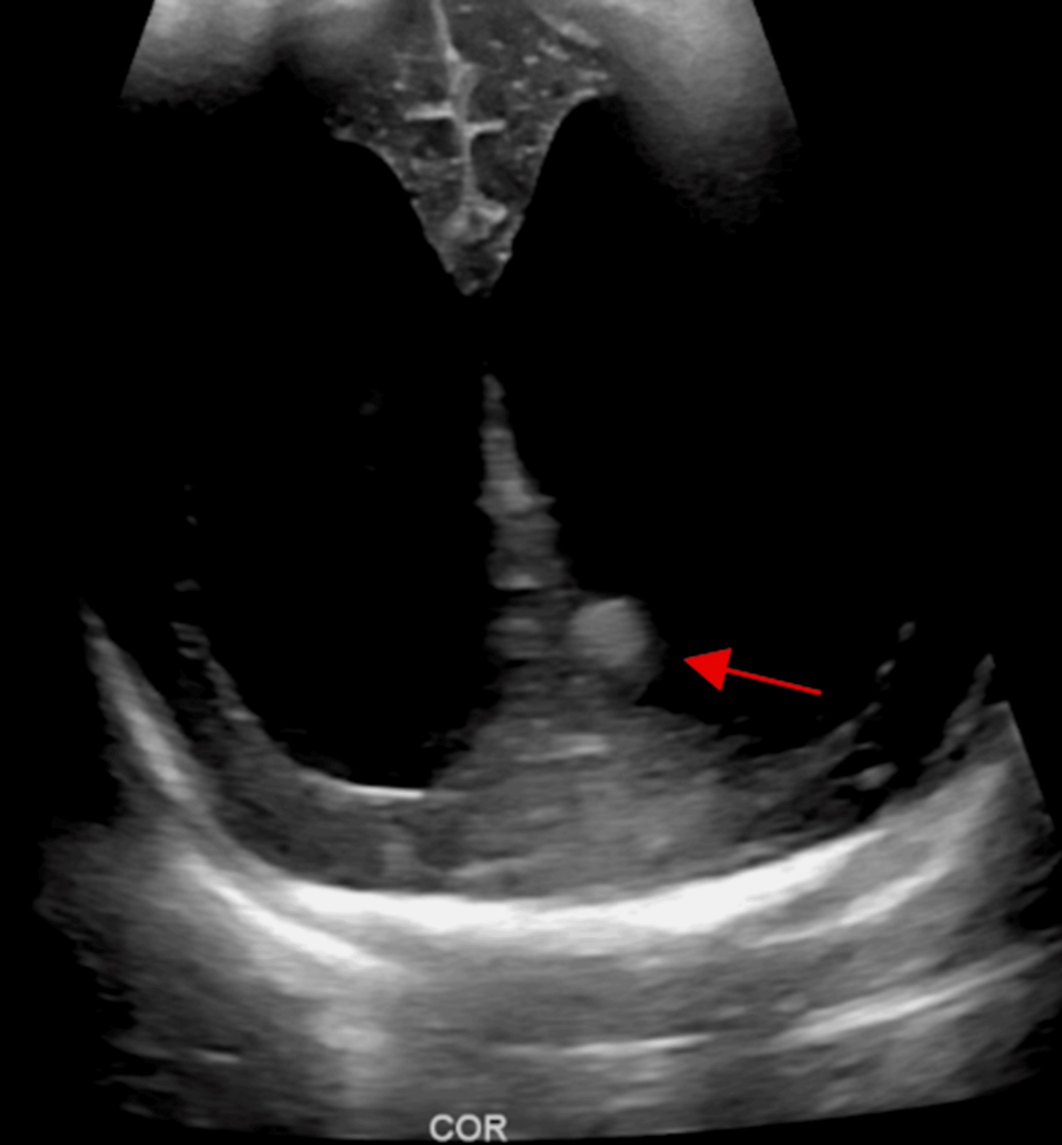
Ultrasonography of twin female with hyperechogenic mass in left lateral ventricle (red arrow) and ventriculomegaly of the lateral ventricles.

To further assess their hydrocephalus, the twins underwent neonatal head ultrasonography (US). In the male, US found severe enlargement of the lateral ventricles without visualization of posterior fossa structures and limited visualization of midbrain structures (Figure 1). In the female, US revealed moderate ventriculomegaly with the batwing configuration of the lateral ventricles (Figure 2) consistent with Chiari 2 malformation. Additionally, the US in the female twin illustrated a small focus of increased echogenicity in the left lateral ventricle consistent with a hemorrhage and small posterior fossa structures (Figure 3).

**Figure 4:**
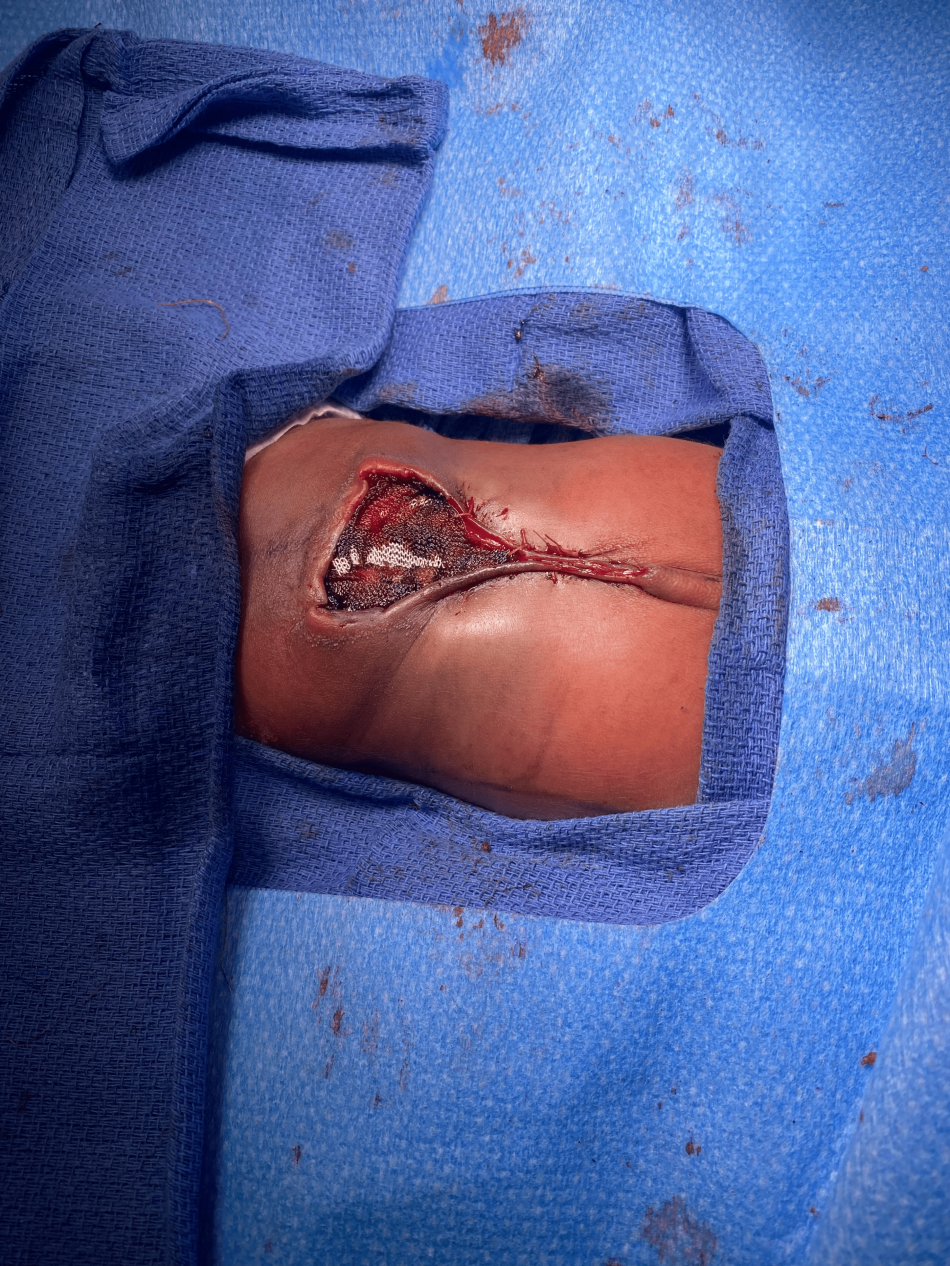
Twin male following neurosurgical and plastic surgery intervention of repair of meningomyelocele.

The neonates underwent successful neurosurgical repair of the meningomyelocele at around 48 hours after birth which involved dissection of the neural placode with tenotomy scissors. In the male, an abnormally shaped lamina was found with no defect in the integrity of the lamina. Following the removal of the placode, plastic surgery was then consulted for closure which included the creation of a right paraspinous flap and a left para spinous flap. Only the superior portion of the defect was able to be closed during initial surgical intervention due to the risk of respiratory complication, therefore the soft tissue defect was closed temporarily with a wound vacuum-assisted closure (VAC) until further intervention with a skin graft could be accomplished (Figure 4).

**Figure 5:**
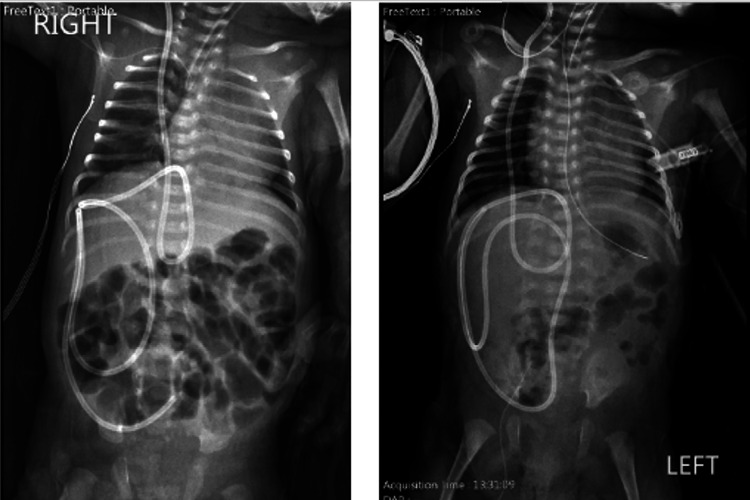
Abdominal x-ray in twins (right=male, left= female) illustrating the placement of ventriculoperitoneal shunts to manage hydrocephalus.

In accordance with the current criteria, a ventriculoperitoneal (VP) shunt was indicated three weeks after birth in both the male and female once they weighed greater than 2,000 g. The 2,000 g weight requirement is utilized to ensure that the neonates were responding and tolerating feeding without any signs of necrotizing enterocolitis. Following the placement of the VP shunts, both twins underwent an abdominal x-ray confirming the placement of the VP shunts (Figure 5).

Due to the extent of the ventriculomegaly, evaluation of further cerebral malformations was not able to be concluded. The twins remained in the neonatal intensive care unit (NICU) to allow all surgical sites to heal and for careful assessment of neurological status during their hospitalization. At the time of discharge, both infants were hemodynamically stable and were scheduled for continuous outpatient monitoring.

## Discussion

Meningomyelocele, also known as open spina bifida is a congenital malformation of the central nervous system associated with significant morbidity and mortality. It is characterized by protrusion of the meninges and spinal cord through an open vertebral arch. Furthermore, patients with meningomyeloceles often have hydrocephalus, bowel and bladder dysfunction, and orthopedic disabilities [4].

Although neural tube defects occur sporadically, several risk factors have been linked to the development of meningomyeloceles including genetic conditions and are more common in twin pregnancies. These genetic conditions can include parents or siblings with a neural tube defect, trisomies 18 and 13, vertebral defects, anal atresia, cardiac defects, tracheo-esphogeal fistula, renal anomalies, and limb abnormalities (VACTERAL association) and X-linked neural tube defects [5]. Additionally, neural tube defects have been associated with Meckel-Gruber syndrome, with typical manifestations of occipital encephalocele, bilateral polycystic kidneys, and post-axial polydactyly [5]. Maternal factors can also play a major role, including exposure to alcohol, caffeine, smoking, polycyclic aromatic hydrocarbons, and hyperthermia or maternal fever in the first trimester. Medical conditions in the mother including elevated glycemic index, gestational diabetes, and nutritional deficiencies are also believed to play a role [4,5]. Folate deficiency, especially during twin pregnancies is a major risk factor for the formation of neural tube defects.

In this case report, we present a set of dizygotic twins with meningomyeloceles born in the United States. Neural tube defects develop in greater frequency in twin gestations; however, it is rare for them to present in both fetuses and a dizygotic gestation. One study reported twins with concordant neural tube defects found only when they occurred as part of a congenital syndrome, and only in same-sexed twins [6] Among those reported to have neural tube defects, the incidence of meningomyeloceles is less in twins compared to anencephaly, and more typically found in non-identical twins [6,7]. Based on a review of the previous research, this case report illustrates the rare occurrence of meningomyeloceles in dizygotic twins.

The diagnosis of meningomyeloceles can be made prenatally via maternal screenings including serum levels of alpha-fetoprotein, however, the screening test of choice is a second-trimester ultrasound [1-3,5]. A positive screening test, as was the case in this pregnancy, requires further evaluation with a complete anatomy scan and genetic fetal karyotype testing to confirm the diagnosis. Following confirmation of the diagnosis, management options include termination of pregnancy, postnatal surgery, or fetal surgery. Of the management options, fetal surgery has been shown to have better outcomes than postnatal repair. Fetal surgery has been shown to decrease the need for shunt placement and is associated with improved mental development and motor function at 30 months [7].

Patients with neural tube defects have an increased incidence of morbidity and mortality the higher the lesion occurs. Further complications associated with meningomyeloceles also include seizures, cognitive impairments, paralysis, neurogenic bladder and bowel dysfunction, and pressure ulcers due to sensory loss [1,4,5].

Overall, once the diagnosis of a neural tube defect is confirmed, paternal education and genetic testing are crucial to improve patient outcomes. Although the diagnosis was made during prenatal testing in this case report, fetal surgery or in-utero intervention was not utilized, ultimately leading to the need for neurosurgical and plastic surgery intervention following uncomplicated delivery in these dizygotic twins.

## Conclusions

This case report serves as an example of dizygotic twins born with meningomyeloceles exhibiting decreased muscle tone in the lower extremities and hydrocephalus requiring neurosurgical intervention. The occurrence of concordant meningomyeloceles in opposite-sex dizygotic twins is extremely rare and raises questions about the etiology of meningomyeloceles. Genetic counseling and research into genetic components are essential to investigate the pathophysiology of meningomyeloceles. Enhanced prenatal screening and fetal MRI should be integrated into the management of high-risk pregnancies to potentially help correct neural tube defects in-utero in an effort to improve quality of life.
